# A life‐time of hazardous drinking and harm to health among older adults: findings from the Whitehall II prospective cohort study

**DOI:** 10.1111/add.15013

**Published:** 2020-03-31

**Authors:** Linda Ng Fat, Steven Bell, Annie Britton

**Affiliations:** ^1^ Research Department of Epidemiology and Public Health University College London London UK; ^2^ British Heart Foundation Cardiovascular Epidemiology Unit, Department of Public Health and Primary Care University of Cambridge, Strangeways Research Laboratory Cambridge UK; ^3^ National Institute for Health Research Blood and Transplant Unit in Donor Health and Genomics at the University of Cambridge, Strangeways Research Laboratory Cambridge UK; ^4^ Stroke Research Group, Department of Clinical Neurosciences Cambridge Biomedical Campus Cambridge UK

**Keywords:** Ageing, binge drinking, biomarkers, cardiometabolic, cardiovascular, hazardous consumption, liver function, mortality

## Abstract

**Aims:**

To investigate associations of life‐time hazardous and binge drinking with biomarkers of cardiometabolic health, liver function, cardiovascular disease (CVD) and mortality.

**Design:**

Prospective cohort study with median follow‐up time to CVD incidence of 4.5 years.

**Setting:**

London, UK: civil servants within the Whitehall II Study.

**Participants:**

A total of 4820 drinkers aged 59–83 years with biological measurements during the 2011–12 survey.

**Measurements:**

Hazardous drinking was defined as having an AUDIT‐C score ≥ 5 calculated at each decade of life, forming the following groups: never hazardous drinker, former early (stopping before age 50), former later (stopping after age 50), current hazardous drinker and consistent hazardous drinker (hazardous drinker at each decade of life).

**Findings:**

More than half the sample had been hazardous drinkers at some point during their life‐time, comprising former early (< age 50) (19%), former later (≥ age 50) (11%), current (21%) and consistent hazardous drinker (AUDIT‐C ≥ 5 across life (5%). After adjusting for covariates, waist circumference was larger with more persistent hazardous drinking (e.g. compared with never hazardous drinkers, former early had increased waist circumference by 1.17 cm [95% confidence interval (CI) = 0.25‐2.08]; former later by 1.88 cm (CI = 0.77–2.98); current by 2.44 cm (CI = 1.50–3.34) and consistent hazardous drinker by 3.85 cm (CI = 2.23–5.47). Current hazardous drinkers had higher systolic blood pressure (2.44 mmHg, CI = 1.19–3.68) and fatty liver index scores (4.05 mmHg, CI = 2.92–5.18) than never hazardous drinkers. Current hazardous drinkers [hazard ratio (HR) = 2.75, CI = 1.44–5.22) had an elevated risk of stroke, and former later hazardous drinkers had an elevated risk of non‐CVD mortality (HR = 1.93, CI = 1.19–3.14) than never hazardous drinkers. Life‐time binge drinking was associated with larger waist circumferences and poorer liver function compared with never binge drinkers.

**Conclusion:**

Hazardous drinking may increase cardiometabolic risk factors; this is made worse by persistent hazardous drinking throughout life, particularly in relation to weight gain, suggesting benefits of early intervention.

## Introduction

Alcohol use disorders, despite common perception, are common among an older population 1, with the number of alcohol‐related admissions which list either the primary or secondary diagnoses as linked to alcohol being highest among 55–74‐year‐olds in the English population 2. There is also concern over how alcohol may be impacting on cardiovascular health at an age where people are at risk of comorbidities and may be more sensitive to the physical effects of alcohol. Research suggests that older drinkers may also not be aware of the risks of their level of consumption 3. Establishing the level of hazardous drinking among older adults and its consequences may enable effective public health interventions to be generated to raise awareness of the effects of alcohol among older adults and ultimately reduce associated harms. Screening tools such as the Alcohol Use Disorders Identification Test (AUDIT), developed by the World Health Organization (WHO), provide a simple method to identify risky drinking providing a ‘framework for intervention to help hazardous and harmful drinkers reduce or cease alcohol consumption’ 4, and are now commonly used in primary care settings.

Alcohol consumption has been found to vary throughout the life‐course 5; taking a single snapshot of consumption may mask the cumulative effects of drinking, including past heavy drinking in individuals currently moderate in their consumption. While there have been several large‐scale observational studies of the association between alcohol consumption and cardiovascular disease (CVD) 6, 7, 8, 9, 10, relatively little is known about how hazardous drinking as defined by the AUDIT‐C is associated with CVD or various biological markers of cardiovascular health. The drinking habits adopted by individuals earlier in life may have long‐term effects, accelerating the age‐of‐onset of disease 11, 12. Capturing the effects of hazardous drinking throughout life may enhance the usefulness of the screening tool, providing further support for reduction at different stages of life.

This study sought to examine associations between life‐time measures of hazardous drinking as classified by responses to the AUDIT‐C and a range of cardiometabolic and liver biomarkers, incident CVD, and mortality. Given that the focus of the study is on a screening tool which is used to identify harmful patterns of alcohol consumption along with the possible health selection effects into non‐drinking 13, 14, the sample is restricted to current drinkers only.

## Methods

### Sample and design

**Figure 1:**
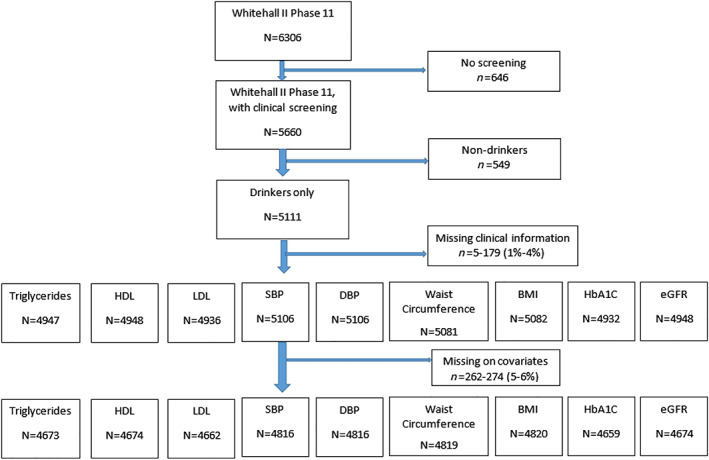
Flow diagram of included participants in analytical sample. BMI = body mass index; DBP = diastolic blood pressure; eGFR = estimated glomerular filtration rate; HbA1C = glycated haemoglobin; HDL = high‐density lipoprotein; LDL = low‐density lipoprotein; SBP = systolic blood pressure. [Colour figure can be viewed at wileyonlinelibrary.com]

This study uses data from the Whitehall II Study, which has been prospectively collecting information from 10 308 (33% female) UK civil servants aged 34–56 years at baseline since 1985–88 15. Data for this study were drawn from the questionnaire and clinical examination stage of Phase 11 collected in 2012–13, when participants were aged between 59 and 83 years (*n* = 6306); 646 participants were not clinically screened. Our sample was further limited to drinkers within the previous year, given the focus of the study on a screening tool used among drinkers to identify harmful patterns of consumption, and health selection effects into former and life‐time non‐drinking 13, 14 (Fig. 1). The final analytical sample was limited to participants with information on all covariates, resulting in approximately 5–6% missing due to item‐non‐response (*n* = 4820–4662). For Cox proportional hazard models, participants with a history of clinically verified CVD were also excluded (*n* = 467).

Ethical approval for the Whitehall II study was received from the University College London Medical School Committee on the ethics of human research, and participants gave written informed consent. Data, protocols and other metadata of the Whitehall II study are available to bona‐fide researchers for research purposes. The Whitehall II data‐sharing policy is available at:https://www.ucl.ac.uk/epidemiology‐health‐care/research/epidemiology‐and‐public‐health/research/whitehall‐ii/data‐sharing

### Life‐time hazardous drinking

A hazardous drinker was defined as someone who scored a total of five or more on the AUDIT‐C; this cut‐off is recognized by Public Health England as being suggestive of an increasing risk drinker 16. The AUDIT‐C involves three questions with five response categories scored from 0 to 5 in order of increasing volume. They cover frequency of drinking (never to four or more times per week), usual number of drinks consumed in a single drinking session (none to two to 10 or more) and number of days drinking heavily (six drinks or more) on a single occasion (never to daily or almost daily). The standard questionnaire can be found elsewhere 17.

Participants were assigned an AUDIT‐C score during each decade of life, from 16–19 to 80+ years, using a retrospective AUDIT‐C life grid method. This information was used to form the following life‐time hazardous drinking categories: never hazardous drinker, former early hazardous drinker (stopped drinking hazardously before age 50), former later hazardous drinker (stopped at age 50 or after), current hazardous drinker, and consistent hazardous drinker (hazardous drinker during every decade of their life) (Table 1 **)**. Age 50 was used as the cut‐off for the two types of former drinkers, given the concern of the rise of substance misuse in older people aged 50 and over 18.

**Table 1:** Categorization of life‐time hazardous drinking groups.

	Hazardous drinker		
	Before age 50	After age 50	Current
Never hazardous drinker	X	X	X
Former early hazardous drinker	√	X	X
Former later hazardous drinker	√ or X	√	X
Current hazardous drinker	√ or X	√	√
Consistent hazardous drinker	√	√	√

X = not a hazardous drinker; √ = hazardous drinker.

As an additional analysis, to distinguish between frequent moderate drinkers who may have scored positive on AUDIT‐C and heavy episodic drinkers, life‐time binge drinking categories were defined using the single item (AUDIT‐3) which asks how often the participant has had six or more drinks on one occasion. Binge drinkers were defined as those who indicated doing so at least monthly.

### Measurement of biomarkers

We examined the effect of life‐time hazardous drinking on objective health, utilizing the clinical measurements taken during the medical examination in Phase 11. These included cardiometabolic biomarkers such as triglycerides (mmol/l), high‐density lipoprotein (HDL) cholesterol (mmol/l), low‐density lipoprotein (LDL) cholesterol (mmol/l), systolic blood pressure (mmHg), diastolic blood pressure (mmHg), waist circumference (cm), body mass index (BMI, kg/m^2^), glycated haemoglobin (HbA1c, mmol/mol) and estimated glomerular filtration rate using serum creatinine and the CKD‐Epi 2009 equation (eGFR, ml/min/1.73 m^2^).

We also examined standard liver function tests including gamma‐glutamyl transferase (GGT, IU/l), alanine aminotransferase (ALT, IU/l), aspartate aminotransferase (AST, IU/l), bilirubin (IU/l) and derived Fatty Liver Index (FLI). The FLI was calculated using a standard algorithm based on triglycerides, GGT, waist circumference and BMI 19, which resulted in an index ranging from 0 to 100, with higher scores reflecting a greater likelihood of having a fatty liver.

### Ascertainment of incident CVD events and mortality

Participants’ data were linked to the national mortality register governed by the National Health Services (NHS) Central register, in which information on mortality was ascertained up to 31 August 2017. Follow‐up for clinically verified fatal and non‐fatal stroke, myocardial infarction (MI), coronary heart disease (CHD) and aggregate CVD was ascertained up to 31 March 2017. Full ICD codes for classification of these diseases can be found elsewhere 20.

### Covariates

Covariates added to each model included sex, age and occupational grade (low, medium, high) in relation to participants’ last known occupation and self‐reported ethnicity (white, non‐white). Health and health behaviours also adjusted for included BMI, self‐reported smoking status (never, ex‐smoker, current smoker), physical activity and fruit and vegetable consumption. Physical activity was dichotomized into whether a participant met or exceeded the WHO recommendation of 2.5 hours of moderate‐to‐vigorous activity per week versus not meeting recommendations. A question relating to frequency of eating fresh fruit and vegetables was categorized into ‘seldom: three to four times week’, ‘daily’ and ‘four or more a day’. We also adjusted for whether participants had a history of any CVD, or previous diagnosis of diabetes based on self‐report at Phase 11 (no/yes).

### Statistical analyses

A series of linear regression models were fitted with each biomarker as an outcome variable and life‐time hazardous drinking groups as the primary exposure, adjusting for all covariates listed above. Models with waist circumference or BMI as the outcome did not adjust for the alternative. The same models were repeated for life‐time binge drinking groups as the primary exposure, which also adjusted for life‐time hazardous drinking (shown in the Supporting information). Where residuals were not normally distributed the outcome variable was natural log‐transformed; this applied to triglycerides, HbA1c, ALT, AST and bilirubin. To ease interpretation of models where the outcome variable was log‐transformed, regression coefficients were multiplied by 100 and interpreted as a percentage difference 21.

The associations between life‐time hazardous or binge drinking and incident CVD and mortality were estimated using Cox proportional hazard models. Survival time was calculated from date of interview in Phase 11 to date of CVD event or censoring (CVD death or last date of data linkage up to 31 March 2017, whichever came first), or to death or censoring (indicating no death up to 31 August 2017). For CVD incidence, median and maximum follow‐up time was 4.5 and 5.3 years. Hazard ratios (HR) and 95% confidence intervals (95% CI) were calculated for each life‐time drinking category, with the never hazardous/binge drinker group treated as reference category. Due to the small number of incident CVD events, current and consistent hazardous drinkers were combined into the one category: ‘current hazardous drinkers’. There was no evidence of deviation from the proportionality assumption in Cox models after assessing Schoenfeld residuals. Survival models were adjusted for the same covariates as the linear regression models with the exception of having a history of CVD, as these participants were excluded from analyses. This paper is an honest, accurate and transparent account of the study being reported; no important aspects of the study have been omitted. These analyses were not pre‐registered; results should be considered exploratory.

## Results

### Sample characteristics

Of 4820 participants (mean age = 69.4 years, 25% female), more than half of drinkers had been hazardous drinkers at some point in their life, with 21% being current hazardous drinkers and 5% being consistent hazardous drinkers (see Table 2). In total, 11% were former early hazardous drinkers and 19% were former later hazardous drinkers. Current and consistent hazardous drinkers were mainly male (80 and 82%, respectively) and predominately white. Those from higher occupational grades were more likely to be current and consistent hazardous drinkers (61%), followed by former later hazardous drinkers (56%), former early hazardous drinkers (53%) and, finally, never hazardous drinkers (44%). The highest proportion of those who experienced a previous CVD event or diabetes diagnoses were former later hazardous drinkers (19%); the lowest was current and consistent hazardous drinkers (13%). The 262 participants who were excluded due to having missing information on covariates were more likely to be female (32%, *P* = 0.022) and non‐white (10%, *P* = 0.001); no other results were significant (Supporting information, Table S1).

**Table 2:** Characteristics of life‐time hazardous drinking groups, Whitehall II study 2011–12.

	Never hazardous drinker		Former early hazardous drinker		Former later hazardous drinker		Current hazardous drinker		Consistent hazardous drinker		Total		*P*‐value
	*n*	%	*n*	%	*n*	%	*n*	%	*n*	%	*N*	%	
All	2110	44	932	19	539	11	1009	21	230	5	4820	100	
Sex													
Male	1347	64	753	81	439	81	838	83	221	96	3598	75	
Female	763	36	179	19	100	19	171	17	9	4	1222	25	*P* < 0.001
Occupational grade													
High	929	44	496	53	303	56	618	61	141	61	2487	52	
Medium	940	45	388	42	213	40	354	35	84	37	1979	41	
Low	241	11	48	5	23	4	37	4	5	2	354	7	*P* < 0.001
Ethnicity													
White	1943	92	914	98	520	96	980	97	228	99	4585	95	
Non‐white	167	8	18	2	19	4	29	3	2	1	235	5	*P* < 0.001
Smoking status													
Never smoker	1189	56	400	43	201	37	336	33	46	20	2172	45	
Ex‐smoker	873	41	500	54	317	59	630	62	168	73	2488	52	
Current smoker	48	2	32	3	21	4	43	4	16	7	160	3	*P* < 0.001
Physical activity													
< recommendations	1483	70	634	68	363	67	673	67	141	61	3294	68	
> = recommendations	627	30	298	32	176	33	336	33	89	39	1526	32	0.033
Fruit and vegetable consumption													
Seldom: 3–4 times a week	430	20	192	21	93	17	227	22	50	22	992	21	
Daily	442	21	170	18	117	22	210	21	45	20	984	20	
4 or more a day	1238	59	570	61	329	61	572	57	135	59	2844	59	0.264
Previous CVD or diabetes	369	17	151	16	100	19	136	13	31	13	787	16	0.022
Mean (SE)													**N**
Age (years)	70.6	(5.87)	67.9	(5.51)	70	(5.50)	68.8	(5.47)	66.2	(4.06)	69.4	(0.46)	4820
Triglycerides (mmol/l)	1.2	(0.55)	1.2	(0.67)	1.2	(0.64)	1.2	(0.74)	1.3	(0.74)	70.4	(0.97)	4673
HDL cholesterol (mmol/l)	1.7	(0.46)	1.5	(0.42)	1.7	(0.49)	1.7	(0.49)	1.6	(0.40)	1.7	(0.46)	4674
LDL cholesterol (mmol/l)	2.9	(0.99)	2.9	(0.96)	2.8	(0.96)	2.9	(0.96)	2.9	(0.97)	2.9	(0.97)	4662
Systolic bp (mmHg)	127.3	(16.55)	126	(15.86)	128	(16.47)	130	(15.97)	129.6	(14.82)	128	(16.26)	4816
Diastolic bp (mmHg)	70.2	(9.62)	70.3	(9.79)	71	(10.12)	72.4	(10.16)	72.9	(9.35)	70.9	(9.86)	4816
Waist (cm)	94.5	(12.34)	96.8	(12.23)	98	(12.03)	98.2	(11.97)	100.5	(11.34)	96.4	(12.29)	4819
BMI (kg/m^2^)	26.4	(4.53)	26.7	(4.49)	27	(4.15)	26.9	(4.15)	27.5	(3.92)	26.7	(4.38)	4820
HbA1c (mmol)	5.9	(0.65)	5.8	(0.64)	5.9	(0.74)	5.7	(0.50)	5.8	(0.52)	5.8	(0.62)	4659
eGFR (ml/min/1.73 m^2^)	77.5	(16.96)	78.8	(15.87)	79	(16.87)	81.6	(16.72)	82.3	(16.70)	79	(16.76)	4674

CVD = cardiovascular disease; HDL = high‐density lipoprotein; LDL = low‐density lipoprotein; BMI = body mass index; eGFR = estimated glomerular filtration rate; SE = standard error; bp = blood pressure; HbA1c = glycated haemoglobin.

**Figure 2:**
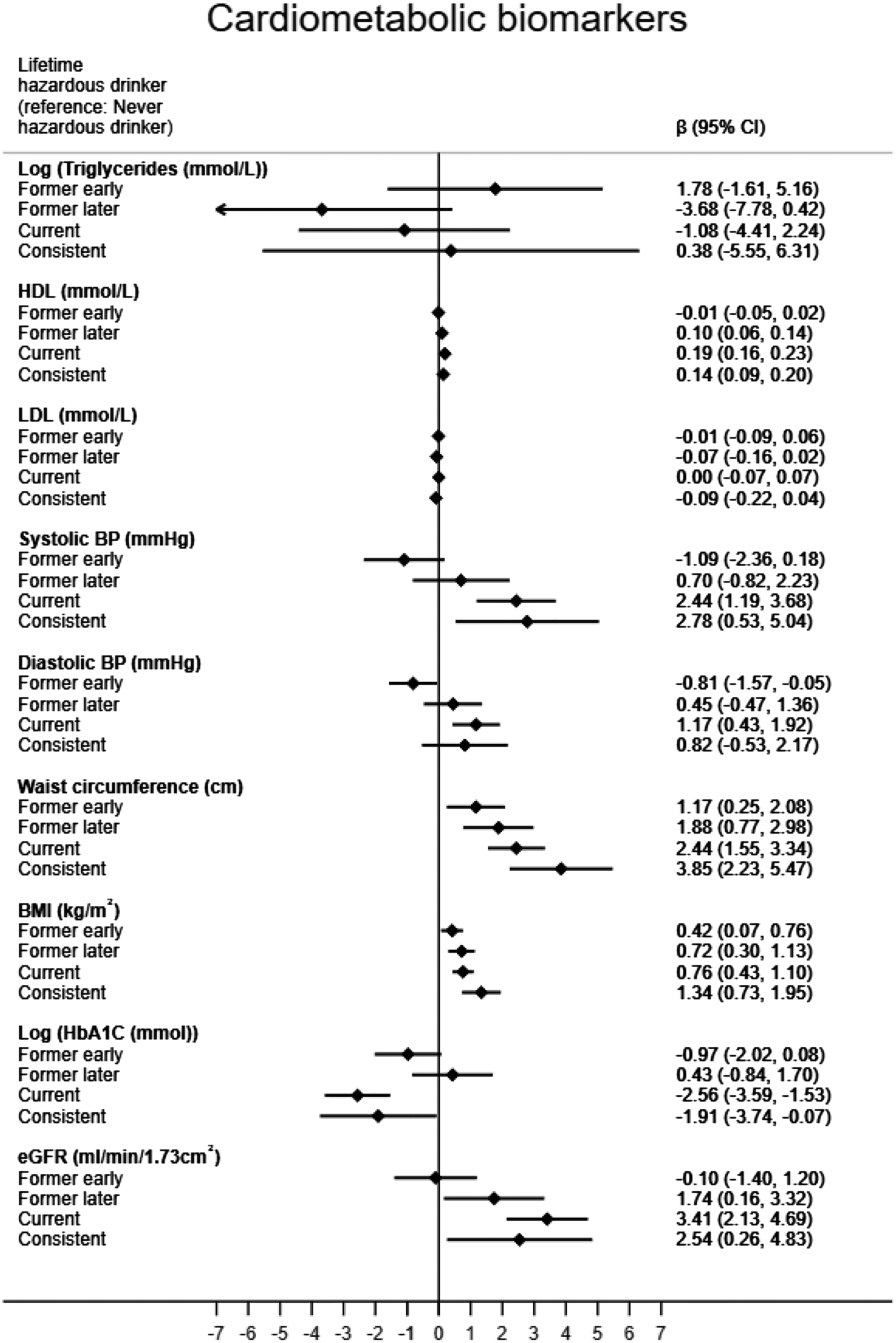
Linear regression results for life‐time hazardous consumption groups and cardiometabolic biomarkers. Adjusted for sex, age, occupational grade, ethnicity smoking status, body mass index (BMI), physical activity, fruit and vegetable consumption and previous cardiovascular disease (CVD) or diabetes diagnosis. Models with BMI or waist circumference as the outcome did not adjust for BMI. eGFR = estimated glomerular filtration rate; HDL = high‐density lipoprotein; LDL = low‐density lipoprotein

Displayed in Fig. 2 are associations between life‐time hazardous drinking and markers of cardiometabolic health. Former later (β = 0.10 mmol/l, 95% CI = 0.06–0.14, *P* < 0.001), current (0.19 mmol/l, 95% CI = 0.16–0.23, *P* < 0.001) and consistent hazardous drinkers (0.14 mmol/l, 95% CI = 0.09–0.20, *P* < 0.001) had significantly higher HDL cholesterol than never hazardous drinkers. Current (2.44 mmHg, 95% CI = 1.19–3.68, *P* < 0.001) and consistent hazardous drinkers (2.78 mmHg, 95% CI = 0.53–5.04, *P* = 0.02) had higher systolic blood pressure than never hazardous drinkers. Current hazardous drinkers also had higher diastolic blood pressure (1.17 mmHg, 95% CI = 0.43–1.92, *P* = 0.02). Results for triglycerides and LDL cholesterol did not reveal any material differences between drinking categories.

Lifetime hazardous drinkers had significantly larger waist circumference and BMI than never hazardous drinkers, with the magnitude increasing with more current and consistent hazardous drinking. For example, for waist circumference, former early hazardous drinkers on average had a 1.17 cm (95% CI = 0.25–2.08, *P* = 0.01) larger waist than never hazardous drinkers, whereas former later hazardous drinkers, current hazardous drinkers and consistent hazardous drinkers had 1.88 cm (95% CI = 0.77–2.98, *P* < 0.01), 2.44 cm (95% CI = 1.55–3.34, *P* < 0.01) and 3.85 cm (95% CI = 2.23–5.47, *P* < 0.01) cm larger waist circumferences, respectively.

Current (−2.56 mmol/mol, 95% CI = −3.59−1.53, *P* < 0.001) and consistent hazardous drinkers (−1.91 mmol/mol, 95% CI = −3.74−0.07, *P* = 0.04) had lower HbA1c values than never hazardous drinkers. Former later (1.74 ml/min/1.73 m^2^, 95% CI = 0.16–3.32, *P* = 0.03), current (3.41 ml/min/1.73 m^2^, 95% CI = 2.13–4.69, *P* < 0.001) and consistent hazardous drinkers (2.54 ml/min/1.73 m^2^, 95% CI = 0.26–4.83, *P* = 0.03) had higher eGFR values than never hazardous drinkers. This relationship maintained for current hazardous drinkers, even with the inclusion of systolic blood pressure; HbA1c and HDL in the model (2.62 ml/min/1.73^2^, 95% CI = 1.33–3.92, *P* < 0.001), however, became non‐significant for consistent hazardous drinkers (1.96 ml/min/1.73^2^, 95% CI = −0.32–4.25, *P* = 0.09) (results not shown).

**Figure 3:**
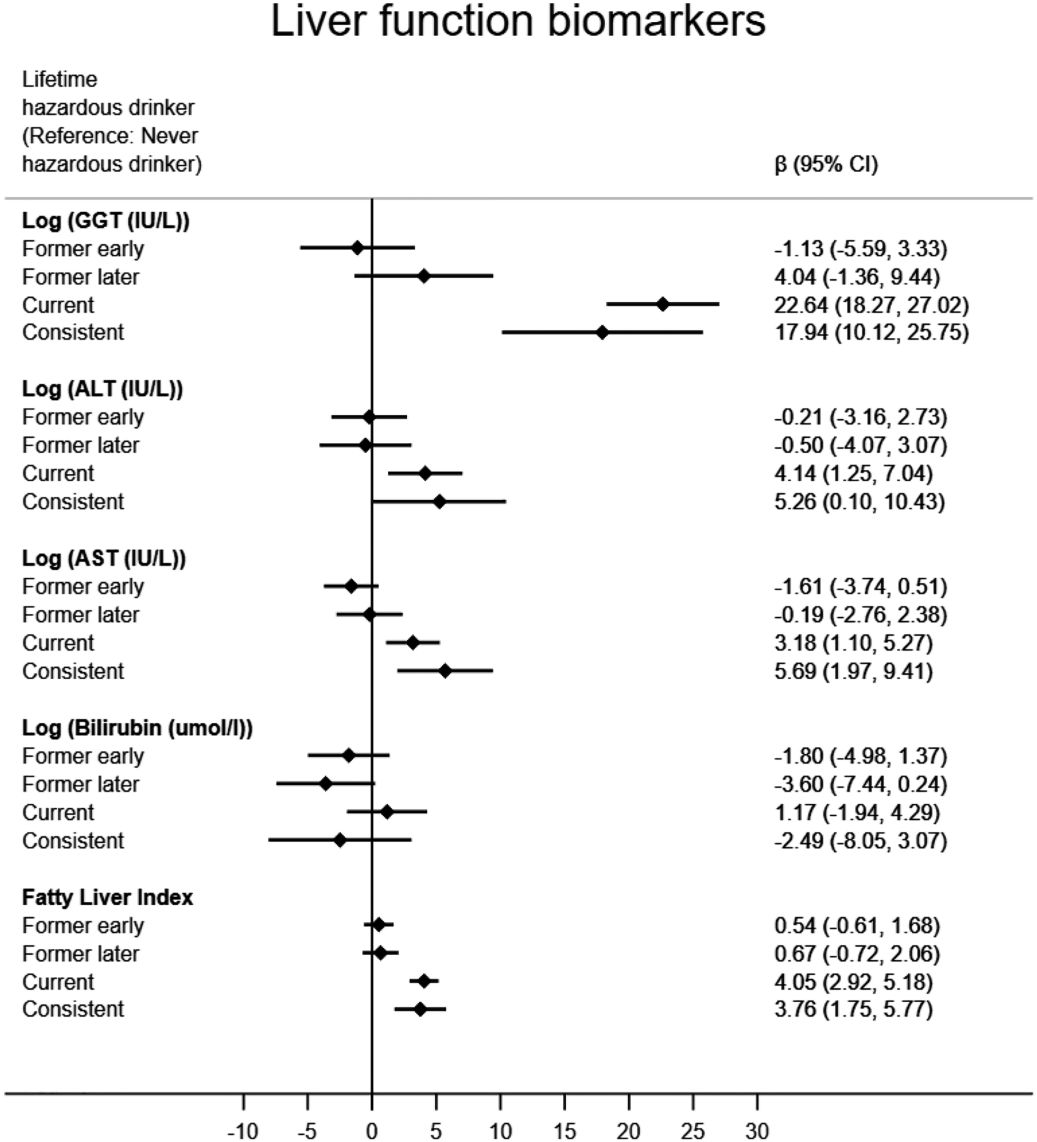
Linear regression results for lifetime hazardous consumption groups and liver function biomarkers. Adjusted for sex, age, occupational grade, ethnicity smoking status, BMI, physical activity, fruit and vegetable consumption and previous cardiovascular disease (CVD) or diabetes diagnosis. ALT = alanine aminotransferase; AST = aspartate aminotransferase; GGT = gamma‐glutamyl transferase

Figure 3 shows similar comparisons between never hazardous drinkers and other life‐time drinking categories among a range of liver function biomarkers. With the exception of bilirubin, current and consistent hazardous drinkers had values indicative of poorer liver function than never hazardous drinkers (GGT; current (22.64 IU/l, 95% CI = 18.27–27.02, *P* < 0.001), consistent (17.94 IU/l, 95% CI = 10.10–25.75, *P* < 0.001), ALT; current (4.14 IU/l, 95% CI = 1.25–7.04, *P* = 0.01), consistent (5.26 IU/l, 95% CI = 0.10–10.43, *P* = 0.04), AST; current (3.18 IU/l, 95% CI = 1.10–5.27, *P* < 0.01), consistent (5.69 IU/l, 95% CI = 1.97–9.41, *P* < 0.01) and FLI; current (4.05, 95% CI = 2.92–5.18, *P* < 0.001), consistent (3.76, 95% CI = 1.75–5.77, *P* < 0.001]. Results for former early and former later hazardous drinkers were non‐significant. As a sensitivity analysis, models with FLI as the outcome that did not adjust for BMI found a greater effect of current (8.39, 95% CI = 6.30–10.48) and consistent hazardous drinking (11.03, 95% CI = 7.29–14.76), as anticipated, but remained in the same direction (results not shown).

Former binge drinkers and current binge drinkers had larger waist circumferences than never binge drinkers. Similarly, all former and current binge drinkers had a greater BMI than never binge drinkers. All other effects failed to reach significance. Current binge drinkers on average had much higher GGT (18.42 IU/l, 95% CI = 11.23–25.61, *P* < 0.001), AST (5.80 IU/l, 95% CI = 2.38–9.23, *P* < 0.01) and FLI scores than never binge drinkers (3.49, 95% CI = 1.65–5.34, *P* < 0.001). Results for consistent binge drinkers failed to reach significance, with the exception of the FLI (4.73, 95% CI = 1.20–8.27) (Supporting information, Table S2)

We observed no statistically significant findings for life‐time hazardous drinking groups and risk of incident MI, any CHD event or aggregate CVD (Table 3). For stroke incidence, current hazardous drinkers had almost a threefold greater risk than never hazardous drinkers (HR = 2.75, 95% CI 1.44–5.22); for the other groups results were not significant. No significant findings were found between life‐time hazardous drinking groups and risk of all‐cause mortality. Former later hazardous drinkers had an approximately twofold higher risk of non‐CVD mortality (1.93, 95% CI = 1.19–3.14) than never hazardous drinkers. Risk of mortality for other groups were non‐significant.

**Table 3:** Associations between lifetime hazardous drinking and incident CVD events and mortality.

	CHD incidence					Stroke incidence				
	No. of cases	Total no.	HR	(95% CI)	*P*‐value	No. of cases	Total no.	HR	(95% CI)	*P*‐value
Never hazardous drinker	88	1881	1.00 (Ref)			18	1881	1.00		
Former early hazardous drinker	41	830	1.10	(0.75, 1.62)	0.615	9	831	1.46	(0.64, 3.31)	0.367
Former later hazardous drinker	24	482	0.91	(0.57, 1.44)	0.686	9	982	1.94	(0.86, 4.38)	0.112
Current hazardous drinker	49	1128	0.85	(0.59, 1.23)	0.399	25	1127	2.75	(1.44, 5.22)	0.002

	MI incidence					CVD incidence				
	No. of cases	Total no.	HR	(95% CI)	*P*‐value	No. of cases	Total no.	HR	(95% CI)	*P*‐value
Never hazardous drinker	20	1881	1.00			111	1182	1.00		
Former early hazardous drinker	14	830	1.50	(0.74, 3.04)	0.266	50	831	1.15	(0.81, 1.62)	0.434
Former later hazardous drinker	6	482	0.92	(0.37, 2.33)	0.864	32	482	1.01	(0.68, 1.51)	0.946
Current hazardous drinker	7	1127	0.46	(0.19, 1.13)	0.091	75	1128	1.13	(0.83, 1.54)	0.445

	Total mortality					Non‐CVD mortality				
	No. of cases	Total no.	HR	(95% CI)	*P*‐value	No of cases	Total no.	HR	(95% CI)	*P*‐value
Never hazardous drinker	75	1893	1.00			56	1893	1.00		
Former early hazardous drinker	28	836	1.14	(0.73, 1.78)	0.573	22	836	1.18	(0.71, 1.96)	0.524
Former later hazardous drinker	26	485	1.48	(0.93, 2.33)	0.095	25	486	1.93	(1.19, 3.14)	0.008
Current hazardous drinker	33	1134	0.94	(0.61, 1.44)	0.771	29	1134	1.10	(0.69, 1.77)	0.687

CVD = cardiovascular disease; CI = confidence interval; HR = hazard ratio; MI = myocardial infarction.

Results for life‐time binge drinkers failed to reach significance (Supporting information, Table S3).

## Discussion

Hazardous drinking was prevalent among this older‐aged cohort, with more than half the participants scoring five or more on the AUDIT‐C at some point in their life. Current and consistent hazardous drinkers had worse liver function, larger BMI and waist circumferences, higher systolic and diastolic blood pressure and an increased risk of stroke compared to never hazardous drinkers, suggesting benefits in these domains from reducing hazardous consumption. Additionally, former hazardous drinkers had larger waist circumferences and BMI and an increased risk of non‐CVD mortality than never hazardous drinkers. Reducing hazardous consumption earlier in life may have positive benefits, in particular with relation to weight gain.

It is well established that higher levels of alcohol consumption increase the risk of liver disease 22. In this respect, our results were as anticipated; current and consistent hazardous had higher values of almost all liver function markers examined in this study. Hazardous drinkers who had quit (either before age 50 or after age 50) did not have worse liver function scores than never hazardous drinkers, which could reflect the ability of the liver to regenerate itself 23. Liver disease is the only major chronic disease to be steadily increasing in the United Kingdom 24 yet, as this study shows, hazardous drinking remains common among an older cohort. Stopping hazardous drinking at any point during the life‐course is likely to be beneficial for liver health; doing so may help to tackle the rapidly growing burden of liver disease in the population.

Larger BMI and waist circumferences were found with more persistent life‐time hazardous drinking, increasing in magnitude with chronicity of being a hazardous drinker, suggesting the benefits of stopping hazardous drinking in relation to weight gain cannot come too early. Other studies have found that life‐time alcohol use (measured in total volume consumed) is associated with higher waist circumference, particularly in men 25. Our results suggest that even hazardous drinking or binge drinking before age 50 can contribute to greater BMI and waist circumference in later life, both of which are major risk factors for heart disease, Type 2 diabetes and cancer 26, 27, 28.

Our finding that current hazardous drinkers have increased blood pressure is consistent with other studies 29, and this might be one pathway implicated in the increased risk of stroke found in this study 30 and elsewhere 8, 31. We also found an association among former later‐life hazardous drinking and increased risk of non‐CVD mortality compared with never hazardous drinkers. This may be due to people who have reduced hazardous drinking in later life due to pre‐existing illness 14, which may be as a result from hazardous consumption.

Relationships with life‐time hazardous drinking in the opposing direction (ie, better for health) were found with HDL, HbA1c and eGFR. The relationship between alcohol consumption and increased HDL levels has been observed in many other studies 6. However, we did not find any significant association between life‐time hazardous drinking and CVD incidence, which is not necessarily surprising; HDL cholesterol may not have a causal role in reducing the risk of myocardial infarction 32. The relationship between alcohol and Type 2 diabetes is likely to be complex, with many previous studies finding reduced risk for moderate drinkers, particularly among women 33. Hazardous alcohol consumption was associated with better renal function as assessed through eGFR. Other studies have found similar results 34, 35, 36, and this counter‐intuitive finding requires further investigation. Our investigation into life‐time hazardous consumption and various cardiometabolic biomarkers highlights the complex relationship between alcohol and health, as drinking appears to affect multiple pathways in different ways.

### Strengths and limitations

The strengths of this study include the ability to explore the effects of hazardous drinking throughout life, where many studies are constrained to assessing alcohol consumption during a short period only. We also assessed hazardous consumption in relation to a range of objective biomarkers of health, providing a comprehensive overview of the effects of alcohol on an array of biological systems, highlighting the complex association of alcohol with health in the process

Our study is, of course, not without limitations. We have used Phase 11 of the study, where subjects have been lost to attrition at every follow‐up since baseline. This is likely to have resulted in a more affluent sample. as those who drop out tend to be from a lower social grade 37. Furthermore, the sample is an occupational cohort and therefore may not be representative of the population 38. This may have resulted in a lower prevalence of disease in an arguably more affluent sample than the general population, and therefore caution should be heeded in generalizing the results. Despite this, associations between risk factors and disease observed in the Whitehall II cohort have been shown to be comparable to those in the general population 39. The ability to explore the association of life‐time alcohol consumption with a range of biomarkers in a relatively large sample arguably balances out this disadvantage. We used a retrospective measure of life‐time hazardous drinking during a long time‐period, which is likely to give rise to recall bias. However, a previous validation study on this measure in Whitehall II showed it to be reliable 40. Similar retrospective measures have been used elsewhere 41, 42 but, as with all self‐reported data, there remains the possibility of bias. Furthermore, due to data constraints there is the possibility of residual confounding, including not accounting for a more comprehensive measure of nutrition. Our short follow‐up period made it difficult to assess the longer‐term effects of hazardous drinking (e.g. we observed only a small number of events), and this might partly explain the many null findings and inability to examine the effects of consistent hazardous drinking in survival models in more detail. Unfortunately, we were not able to split stroke by subtype. Our categorization of life‐time hazardous consumption may have resulted in a loss of information; however, the groups were large enough to facilitate interpretation, in particular the effects of being a hazardous drinker before mid‐life only. Finally, study participants were aged 59 years and over, therefore we may have not captured the full effects of hazardous drinking throughout life, particularly among younger hazardous drinkers who may have dropped out of the study.

## Conclusion

Hazardous drinking, as identified through the AUDIT‐C screening tool, is common among older adults and may increase cardiometabolic risk factors. Population reductions in hazardous drinking are likely to result in improvements in liver function and blood pressure in elderly people and confer a reduced risk of stroke. Longer‐lasting gains in health and wellbeing may accrue with earlier intervention in the life course, particularly in relation to weight gain.

## Declaration of interests

None.

## Supporting information

Table S1 Characteristics of excluded participants due to missing information on variables, compared with sample and chi‐squared tests
Table S2 Associations between cardiometabolic and liver function biomarkers and lifetime binge drinking
Table S3 Associations between lifetime binge drinking and incident CVD events, and Mortality.
