# Supplementary material for: A life‐time of hazardous drinking and harm to health among older adults: findings from the Whitehall II prospective cohort study
Source: Addiction. 2020 Mar 31;115(10):1855–66. doi: 10.1111/add.15013 (PMC7487058; doi:10.1111/add.15013)
Supplement: Supplementary file 1 — Table S1 Characteristics of excluded participants due to missing information on variables, compared with sample and chi‐squared tests Table S2 Associations between cardiometabolic and liver function biomarkers and lifetime binge drinking Table S3 Associations between lifetime binge drinking and incident CVD events, and Mortality. [file ADD-115-1855-s001.docx]

| **Supplementary Table 1** |  | |  | |  | |  |
| --- | --- | --- | --- | --- | --- | --- | --- |
|  | Sample | | Missing | | Total | | p-value |
|  | N | % | N | % | N | % |  |
| N | 4820 |  | 262 |  | 5082 |  |  |
|  |  |  |  |  |  |  |  |
| **Sex** |  |  |  |  |  |  |  |
| Male | 3598 | 75 | 179 | 68 | 3777 | 74 |  |
| Female | 1222 | 25 | 83 | 32 | 1305 | 26 | 0.022 |
|  |  |  |  |  |  |  |  |
| **Occupational grade** |  |  |  |  |  |  |  |
| High | 2487 | 52 | 126 | 48 | 2613 | 51 |  |
| Medium | 1979 | 41 | 107 | 41 | 2086 | 41 |  |
| Low | 354 | 7 | 29 | 11 | 383 | 8 | 0.075 |
|  |  |  | 262 |  |  |  |  |
| **Ethnicity** |  |  |  |  |  |  |  |
| White | 4585 | 95 | 232 | 90 | 4817 | 95 |  |
| Non-white | 235 | 5 | 25 | 10 | 260 | 5 | 0.001 |
|  |  |  |  |  |  |  |  |
| **Smoking status** |  |  |  |  |  |  |  |
| Never smoker | 2172 | 45 | 17 | 45 | 2189 | 45 |  |
| Ex-smoker | 2488 | 52 | 20 | 53 | 2508 | 52 |  |
| Current smoker | 160 | 3 | 1 | 3 | 161 | 3 | 0.969 |
|  |  |  |  |  |  |  |  |
| **Physical activity** |  |  |  |  |  |  |  |
| < recommendations | 3294 | 68 | 183 | 73 | 3477 | 69 |  |
| >= recommendations | 1526 | 32 | 69 | 27 | 1595 | 31 | 0.154 |
|  |  |  |  |  |  |  |  |
| **Fruit & vegetable consumption** |  |  |  |  |  |  |  |
| Seldom-3-4 times a week | 992 | 21 | 47 | 20 | 1039 | 21 |  |
| Daily | 984 | 20 | 55 | 23 | 1039 | 21 |  |
| 4 or more a day | 2844 | 59 | 133 | 57 | 2977 | 59 | 0.540 |
|  |  |  |  |  |  |  |  |
| **Previous CVD or Diabetes** | 787 | 16 | 45 | 17 | 832 | 16 | 0.718 |

| **Supplementary table 2 Associations between cardiometabolic and liver function biomarkers and lifetime binge drinking** | | | | | | | | |  |  | |  |
| --- | --- | --- | --- | --- | --- | --- | --- | --- | --- | --- | --- | --- |
|  | log (Triglycerides) (mmol/L) | N=4673 | HDL (mmol/L) | N=4674 | LDL (mmol/L) | N=4662 | Systolic bp (mm Hg) | N=4816 | Diastolic bp (mm Hg) | | N=4816 | |
|  | β | 95% (CI) | β | 95% (CI) | β | 95% (CI) | β | 95% (CI) | β | | 95% (CI) | |
| Never binge drinker | Ref |  | Ref |  | Ref |  | Ref |  | Ref | |  | |
| Former early binge drinker | 1.14 | (-2.84, 5.11) | -0.03 | (-0.07, 0.01) | 0.03 | (-0.05, 0.12) | -0.26 | (-1.76, 1.23) | -0.03 | | (-0.92, 0.87) | |
| Former later binge drinker | 3.96 | (-2.07, 9.99) | 0.02 | (-0.04, 0.07) | 0.03 | (-0.09, 0.16) | 0.23 | (-2.03, 2.49) | -0.65 | | (-2.01, 0.70) | |
| Current binge drinker | 2.44 | (-3.04, 7.91) | 0.05 | (0.00, 0.10) | 0.07 | (-0.05, 0.19) | -0.35 | (-2.42, 1.72) | 0.34 | | (-0.90, 1.58) | |
| Consistent binge drinker | 2.56 | (-7.90, 13.02) | -0.01 | (-0.11, 0.08) | -0.15 | (-0.37, 0.078) | 0.86 | (-3.12, 4.83) | 0.65 | | (-1.73, 3.03) | |
|  | Waist (cm) | N=4819 | BMI (kg/m^2^) | N=4820 | Log(Hba1C (mmol)) | N=4659 | eGFR (mL/min/1.73m^2^) | N=4693 |  | |  | |
|  | **β** | 95% (CI) | β | 95% (CI) | β | 95% (CI) | β | 95% (CI) |  | |  | |
| Never binge drinker | Ref |  | Ref |  | Ref |  | Ref |  |  | |  | |
| Former early binge drinker | **1.83** | **(0.76, 2.91)** | **0.71** | **(0.31, 1.11)** | -0.82 | (-2.05, 0.41) | 0.21 | (-1.32, 1.74) |  | |  | |
| Former later binge drinker | 0.93 | (-0.69, 2.56) | **0.79** | **(0.18, 1.40)** | -1.11 | (-2.97, 0.76) | 0.05 | (-2.27, 2.38) |  | |  | |
| Current binge drinker | **2.67** | **(1.18, 4.15)** | **0.88** | **(0.33, 1.44)** | -0.89 | (-2.59, 0.80) | 1.78 | (-0.32, 3.89) |  | |  | |
| Consistent binge drinker | 2.75 | (-0.12, 5.62) | 0.49 | (-0.58, 1.56) | 2.03 | (-1.20, 5.27) | 1 | (-3.03, 5.02) |  | |  | |
|  | Log (GGT) (IU/L) | N=4674 | Log (ALT) (IU/L) | N=4674 | Log (AST) (IU/L) | N=4674 | log(Bilirubin) (umol/l) | N=4674 | Fatty Liver Index | | N=4665 | |
|  | β | 95% (CI) | β | 95% (CI) | β | 95% (CI) | β | 95% (CI) | β | | 95% (CI) | |
| Never binge drinker | Ref |  | Ref |  | Ref |  | Ref |  | Ref | |  | |
| Former early binge drinker | -1.78 | (-7.00, 3.45) | 0.45 | (-3.01, 3.91) | -1.27 | (-3.76, 1.22) | -2.43 | (-5.87, 1.01) | 0.11 | | (-1.23, 1.46) | |
| Former later binge drinker | 1.08 | (-6.84, 9.01) | -1.98 | (-7.23, 3.28) | 0.66 | (-3.12, 4.43) | -3.44 | (-7.71, 0.82) | -0.77 | | (-2.81, 1.26) | |
| Current binge drinker | **18.42** | **(11.23, 25.61)** | 4.32 | (-0.44, 9.08) | **5.8** | **(2.38, 9.23)** | 1.52 | (-2.09, 5.12) | **3.49** | | **(1.65, 5.34)** | |
| Consistent binge drinker | 12.88 | (-0.86, 26.62) | -1.31 | (-10.41, 7.80) | 2.43 | (-4.12, 8.98) | -2.56 | (-9.62, 4.50) | **4.73** | | **(1.20, 8.27)** | |

Adjusted for sex, age, occupational grade, ethnicity smoking status, BMI, physical activity, fruit & vegetable consumption, and previous CVD or diabetes diagnosis. Models with BMI or Waist circumference as the outcome did not adjust for BMI

| **Supplementary Table 3 Associations between lifetime binge drinking and incident CVD events, and Mortality** | | | | | | | | | | | | |
| --- | --- | --- | --- | --- | --- | --- | --- | --- | --- | --- | --- | --- |
|  | **CHD incidence** | | | | |  | **Stroke incidence** | | | | | |
|  | **No of cases** | **Total No** | **HR** | **95% (CI)** | **p-value** |  | **No of cases** | **Total No** | **HR** | **95% (CI)** | **p-value** |  |
| Never binge drinker | 143 | 3100 | 1.00 (Ref) |  |  |  | 39 | 3100 | 1.00 |  |  |  |
| Former early binge drinker | 24 | 606 | 0.84 | (0.52, 1.37) | 0.488 |  | 6 | 606 | 0.71 | (0.28, 1.79) | 0.470 |  |
| Former later drinker | 17 | 219 | 1.42 | (0.79, 2.56) | 0.242 |  | 7 | 219 | 1.57 | (0.63, 3.96) | 0.335 |  |
| Current binge drinker | 18 | 396 | 1.11 | (0.61, 2.02) | 0.735 |  | 9 | 396 | 1.30 | (0.55, 3.05) | 0.552 |  |
|  |  |  |  |  |  |  |  |  |  |  |  |  |
|  | **MI incidence** | | | | |  | **CVD incidence** | | | | | |
|  | **No of cases** | **Total No** | **HR** | **95% (CI)** | **p-value** |  | **No of cases** | **Total No** | **HR** | **95% (CI)** | **p-value** |  |
| Never binge drinker | 36 | 3099 | 1.00 |  |  |  | 188 | 3102 | 1.00 |  |  |  |
| Former early binge drinker | 5 | 606 | 0.55 | (0.20, 1.50) | 0.240 |  | 29 | 606 | 0.76 | (0.50, 1.18) | 0.221 |  |
| Former later drinker | 3 | 219 | 0.95 | (0.24, 3.75) | 0.943 |  | 24 | 219 | 1.45 | (0.88, 2.37) | 0.143 |  |
| Current binge drinker | 3 | 396 | 1.20 | (0.27, 5.29) | 0.814 |  | 27 | 396 | 1.10 | (0.67, 1.78) | 0.715 |  |
|  |  |  |  |  |  |  |  |  |  |  |  |  |
|  |  |  |  |  |  |  |  |  |  |  |  |  |
|  | **Total mortality** | | | | |  | **Non-CVD mortality** | | | | | |
|  | **No of cases** | **Total No** | **HR** | **95% (CI)** | **p-value** |  | **No of cases** | **Total No** | **HR** | **95% (CI)** | **p-value** |  |
| Never binge drinker | 118 | 3118 | 1.00 |  |  |  | 91 | 3118 | 1.00 |  |  |  |
| Former early binge drinker | 21 | 609 | 1.12 | (0.66, 1.90) | 0.670 |  | 20 | 609 | 1.36 | (0.78, 2.37) | 0.278 |  |
| Former later drinker | 10 | 220 | 0.97 | (0.47, 2.02) | 0.937 |  | 10 | 220 | 1.19 | (0.56, 2.51) | 0.646 |  |
| Current binge drinker | 13 | 401 | 1.40 | (0.69, 2.85) | 0.350 |  | 11 | 401 | 1.44 | (0.67, 3.11) | 0.353 |  |
| Participants with clinically verified CVD were excluded from analyses (n=467). Adjusted for sex, age, occupational grade, lifetime hazardous consumption, ethnicity, smoking status, BMI, physical activity, fruit & vegetable consumption. | | | | | | | | | | | | |
